# Air and Bone-Conduction Masseteric Vestibular Evoked Myogenic Potentials: A Simulated Conductive Hearing Loss Study

**DOI:** 10.1055/a-2846-4560

**Published:** 2026-08-14

**Authors:** Raghav Katyal, Jim Saroj Winston

**Affiliations:** 1 Nitte Institute of Speech and Hearing, K S Hegde Medical Academy, Nitte (Deemed to be University), Mangalore, Karnataka, India

**Keywords:** Masseter VEMP, conductive hearing loss, bone conduction, vestibular evoked myogenic potential, occlusion effect

## Abstract

**Introduction:**

Masseteric vestibular evoked myogenic potentials assess saccular-masseteric pathway function using air-conducted or bone-conducted stimuli.

**Objective:**

This study investigated whether simulated bilateral conductive hearing loss differentially affects mVEMP response characteristics under air- and bone-conducted stimulation in young adults.

**Methods:**

Fifty healthy participants were included in the study. Conductive hearing loss was simulated in 25 individuals using silicone-blocked foam ear tips to create controlled bilateral attenuation. Masseteric vestibular evoked myogenic potentials were elicited using 500 Hz tone bursts delivered to the right ear through air and bone-conducted methods. Responses were recorded using a two-channel zygomatic electrode placement and analyzed for P1 and N1 latencies and P1-N1 peak-to-peak amplitude.

**Results:**

In the simulated conductive hearing loss group, air-conducted masseteric vestibular evoked myogenic potential responses showed decreased response rate, delayed latency, and reduced amplitude compared with normal hearing participants. In contrast, bone-conducted masseteric vestibular evoked myogenic potentials were consistently recorded in both groups, demonstrating significantly higher amplitudes and earlier latencies than air-conducted responses in the simulated conductive hearing loss group. Spectral analysis revealed a consistent peak around 100 Hz for all conditions except air-conducted stimulation in the simulated conductive hearing loss group, which showed no distinct peak.

**Conclusion:**

Simulated conductive hearing loss significantly affects air-conducted masseteric vestibular evoked myogenic potential responses, often rendering them absent or diminished. However, bone-conducted responses remain robust and reliable in normal and simulated conductive hearing loss conditions, highlighting their diagnostic value when conductive pathologies compromise air-conducted responses.

## Introduction

The vestibular-evoked myogenic potential (VEMP) has become a critical tool in evaluating vestibular reflex pathways. While cervical VEMP (cVEMP) and ocular VEMP (oVEMP) are widely recognised for assessing the integrity of otolith organs and their associated pathways, 1 the masseteric VEMP (mVEMP) represents a relatively recent and increasingly investigated addition to this family. mVEMPs are recorded from the masseter muscle and are thought to reflect the function of the saccule-masseteric pathway , which may play a role in head stabilisation during motor functions such as chewing. 2 A unique physiological characteristic of the mVEMP pathway is its bilateral projections, contrasting with the ipsilateral projections of cVEMP and the contralateral projections of oVEMP. 2 3 Masseteric VEMP (mVEMP) responses have been implicated in a range of vestibular pathologies , including peripheral conditions like vestibular neuritis, 4 and central pathologies such as brainstem lesions, Parkinson's disease, 5 and amyotrophic lateral sclerosis. 6

Traditionally, VEMPs are elicited via air conduction (AC) stimulation using calibrated insert earphones. 7 However, the AC VEMP is susceptible to various procedure-related factors, such as stimulus type 8 and intensity, 9 as well as subject-related variables, such as age and gender. 9 10 A significant challenge for AC VEMP elicitation is the presence of middle ear conductive pathologies, which impair the efficient transmission of AC stimuli to the inner ear. 11 12 13 Such conductive hearing loss (CHL) can result in considerable attenuation of sound intensity, leading to prolonged latencies, reduced amplitudes, or even absent responses. 12 13 Consequently, bone-conducted (BC) VEMPs have emerged as a suggested alternative stimulation method.

BC stimulation offers a critical advantage by bypassing the middle ear transmission route, directly stimulating the otolith organs. 14 While BC VEMPs have been extensively studied for cVEMP and oVEMP, studies specifically exploring BC stimulation of the saccule-masseteric pathway remain scarce. A recent study highlighted that AC mVEMP responses were absent in the presence of CHL when using 500 Hz tone burst stimuli at 96 dBnHL, whereas BC mVEMP responses were consistently present in all 10 CHL ears. 13 Given the inherent heterogeneity of CHL, with its diverse etiologies and variable effects on middle ear characteristics, 14 conducting well-controlled studies on its impact on VEMP can be challenging. To mitigate this, the present study employed a simulation approach by occluding the external auditory meatus with deeply inserted silicone-blocked foam inserts. 15 This method effectively blocks the AC pathway while preserving the BC pathway, closely mimicking the conditions of CHL and allowing for a controlled investigation.

Within this controlled context, the study specifically investigated the differential effects of simulated CHL on AC- and BC-elicited mVEMP latencies and amplitudes. Additionally, the modulation of ipsilateral and contralateral mVEMP responses to AC and BC stimulation under simulated CHL was examined. An important objective was also to analyze the spectral composition of the recorded mVEMP waveforms in each condition using frequency-domain techniques such as Fast Fourier Transform (FFT). Spectral analysis of the recorded responses provides a complementary perspective to conventional time-domain measures by capturing underlying frequency components and offering a more detailed understanding of response morphology and muscle activation characteristics. Prior studies have highlighted the relevance of such analyses in differentiating true neurogenic signals from noise and in better characterizing pathological alterations in evoked potentials. 16 17 Insights from this spectral evaluation, in conjunction with traditional parameters, can enhance the diagnostic precision of BC mVEMPs in CHL populations and contribute to developing more refined clinical assessment protocols.

## Materials and Methods

### Participants

This study employed a cross-sectional study design. Fifty undergraduate and postgraduate female student volunteers (mean age: 21.04 +- 2.05 years), from the parent university, participated in the study. Only female participants were included to avoid confounding effects related to sex-based variability. A detailed pre-simulation audiological evaluation indicated that all participants' pure-tone audiometry thresholds were within 15 dBnHL for AC and BC stimulation at standard audiometric test frequencies with an air-bone gap of less than 10 dB. Normal middle ear and outer hair cell status were confirmed by the presence of a Type-A tympanogram and transient evoked otoacoustic emissions (TEOAEs) in all participants. Further, all participants performed normally on a modified Clinical Test of Sensory Interaction on Balance (mCTSIB). Individuals previously diagnosed with otologic, neurologic, systemic, or metabolic conditions and those with a history of noise exposure or ototoxic drug usage were excluded from the study. No additional inclusion or exclusion criteria were applied.

### Procedure

All test procedures were conducted in an acoustically and electrically shielded environment compliant with ANSI S3.1-1999 standards for ambient noise in the audiometric test room. 18

#### Audio- Vestibular Evaluations

An Inventis Piano audiometer (Inventis Inc., Padova, Italy) with ER3A insert earphones (Etymotic Research, Elk Grove Village, IL, USA) and a B-71 bone vibrator was used for pure-tone audiometry. The modified Hughson-Westlake procedure 19 was used to estimate the bilateral pure-tone thresholds for BC and AC at octave frequencies between 250 Hz and 8000 Hz for AC and 250 Hz and 4000 Hz for BC. Middle ear status was evaluated using an Inventis Clarinet immittance meter (Inventis Inc.) by presenting a standard 226-Hz probe tone at 80 dB SPL. All participants had a type 'A' tympanogram, suggesting normal middle ear functioning. An Otodynamics ILO Analyser (Otodynamics Inc., USA) was used to record the TEOAEs elicited for click stimuli delivered at 80 dB SPL. TEOAEs were considered present if the SNR at three adjacent test frequencies was above 6 dB. Robust TEOAEs were obtained for all the participants. The mCTSIB was performed on a Postura Stabilometry Platform (Cyclops MedTech, India). The participants' sensory motor integration was assessed while standing on a firm surface and a foam surface under closed and open eyes conditions. A mean centre of pressure sway velocity of ≤ 0.3°/sec on the firm surface and ≤ 0.5°/sec on the foam surface was considered within the normative range.

### Simulation of Conductive Hearing Loss

**Fig. 1:**
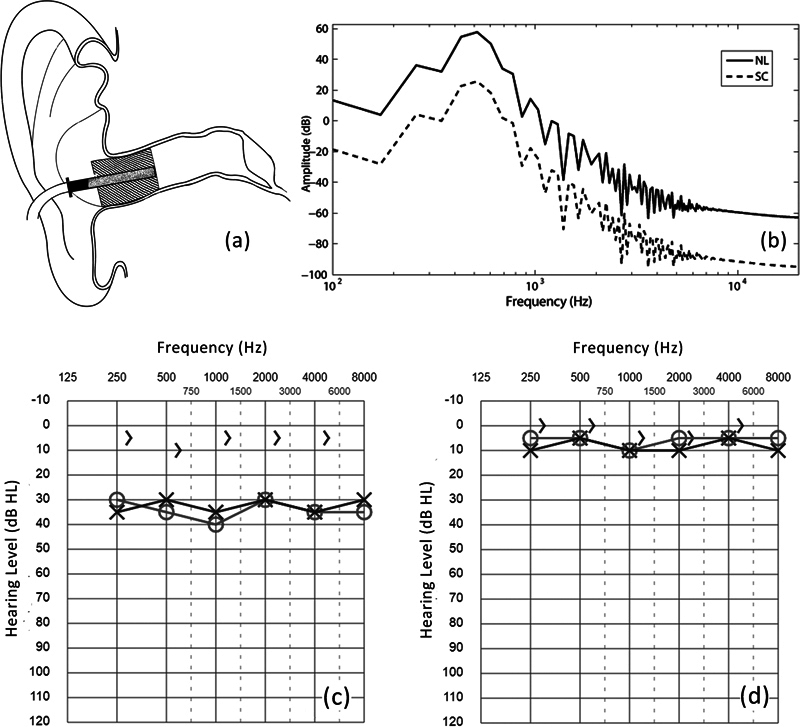
( **A** ) Schematic representation of the simulation procedure, ( **B** ) expected impact of the simulation on the stimulus power spectrum, (C) post-simulation audiogram from one representative participant in the SC group depicting the bilateral mild conductive hearing loss and ( **D** ) normal audiogram from a subject in the normal (NL) group.

From the 50 participants, 25 were randomly assigned to the simulated CHL (SC) group. To simulate conductive hearing loss, a foam ear tip (13 mm; Etymotic Research Inc., USA) was occluded using silicone material (Detax GmbH & Co., Germany) ( Fig. 1 (a) ). This method ensures a controlled and consistent attenuation across all the participants, creating a reliable model for studying SC's impact on vestibular function ( Fig. 1 (b) ). Pure-tone audiometry was repeated with the blocked foam tip in both ears of the simulated SC group to determine and document its effect on the thresholds in AC and BC modalities. The audiometric findings confirmed that the occluded foam tip simulated mild bilateral conductive hearing loss ( Fig. 1 (c & d) ).

### Recording of mVEMP

mVEMP was recorded from all participants in the NL and SC groups using the VEMP module of the Intelligent Hearing Systems (IHS) SmartEP system (IHS, Miami, FL, USA) with electromyography (EMG) monitoring. The 500-Hz tone burst stimulus for AC mVEMPs was delivered using Etymotic Research ER3C insert earphones, and for BC mVEMPs using a B-71 bone vibrator, both in rarefaction polarity. For AC mVEMP, the stimulus was delivered at 96 dBnHL to the right ear; for BC mVEMP, the stimulus was delivered at 68 dBnHL using an envelope of 2-1-2 cycles, and the bone vibrator was placed firmly over the right mastoid with consistent coupling pressure throughout the recording.

The participants were asked to sit comfortably on a chair in a quiet room with a straight, upright posture. The electrode placement sites were identified and prepared to reduce the impedance (<5kΩ). The ground electrode was placed on the participant's forehead, the reference electrodes bilaterally at the midpoint of the zygomatic arch, and the active electrodes bilaterally over the lower third of the masseter muscle. 20 Participants were instructed to clench their posterior teeth to activate the masseter muscle while muscle tension was monitored and maintaining the desired levels on the EMG monitor. Each participant was provided a rest period for two minutes after each recording to avoid muscle fatigue. The analysis time window was 50 msec and 100 msec post-stimulus, with 300 sweeps recorded and EMG monitoring kept between 30 and 150 µV. The response elicited by 300 sweeps of 500-Hz tone bursts was filtered between 1 and 1,500 Hz and amplified 5,000 times. The resulting waveforms were subjected to analysis to identify the landmarks.

### Response Analysis

Two experienced audiologists independently reviewed the recordings, carefully marking P1 and N1 peaks in each resultant waveform. The absolute latencies of each peak and the P1-N1 peak-to-peak amplitude were noted. The values were recorded separately for ipsilateral and contralateral responses, allowing for a detailed assessment of response symmetry. Statistical analysis was performed using JASP (version 0.16.4.0; Love et al., 2019). 21 The Shapiro-Wilk test was used to test data compliance with the normality assumption. Since the data violated the normality assumption (Shapiro-Wilk test, p < 0.05), non-parametric tests were

employed for between-group comparisons. Specifically, the Mann-Whitney U test was used to assess differences in audiometric thresholds and mVEMP parameters between the NL and SC groups. The Wilcoxon signed-rank test was also used to compare AC and BC mVEMP within groups. The power spectrum of the grand average waveforms of each condition was subjected to the Fast Fourier transform. The power spectrum was extracted and plotted using a code running on MATLAB Online (MathWorks).

## Results

### Impact of Simulated Conductive Hearing Loss on Audiometric Thresholds

In the NL group, AC pure-tone averages (PTAs) were 9.13 dB HL (SD = 1.88) in the right ear and 8.97 dB HL (SD = 2.06) in the left. The SC group had significantly higher AC thresholds: 33.00 dB (SD = 4.89) (right) and 31.60 dB (SD = 5.03) (left). BC PTAs were similar between groups: 6.17 dB HL (SD = 0.88) in the NL group vs. 6.04 dB HL (SD = 0.89) in the SC group. Shapiro-Wilk tests indicated non-normal distributions (p < 0.05). Mann-Whitney U tests confirmed significant AC threshold differences between groups (right ear: U = 0.00, p < 0.001, r_rb = -1.00; left ear: U = 0.00, p < 0.001, r_rb = -1.00) , but no BC threshold difference (U = 337.50, p = 0.63, rrb = 0.08).

### Air Conduction mVEMP- Between Group Comparison

In AC mVEMP recordings, all 25 NL participants showed clear responses (100% response rate), while only 6 of 25 SC participants had identifiable responses (24%). Due to unequal sample sizes, statistical results should be interpreted cautiously to avoid Type II errors or effect size inflation.

Among the six SC participants, P1-N1 latencies were prolonged and amplitudes reduced compared to the NL group ( Table 1 ). Mann-Whitney U tests revealed significantly prolonged P1 (U = 150.00, p < 0.001, rrb = 1.00) and N1 latencies (U = 147.00, p < 0.001, rrb = 1.00) and reduced P1-N1 amplitudes (U = 0.0, p < 0.01, rrb = -1.00) ipsilaterally in the SC group. Contralaterally, P1 (U = 150.00, p < 0.001, rrb = 1.00) and N1 (U = 144.00, p < 0.001, rrb = -1.00) latencies were also delayed, with significantly lower amplitudes (U = 0.00, p < 0.001, rrb = -1.00) ( Table 1 ).

**Table 1:** Descriptive statistics of AC and BC mVEMP latency and amplitude parameters in the NL and SC groups.

		SC				NL		
Stimulation Modality	Ipsi vs Contra	Measures	Mean ± SD	Median	IQR	Mean ± SD	Median	IQR
BC	Ipsi	P1 Latency (ms)	15.28 ± 0.25	15.40	0.20	15.34 ± 0.25	15.50	0.20
		N1 Latency (ms)	19.87 ± 0.50	20.10	0.80	20.90 ± 0.68	20.90	0.90
		P1-N1 Amplitude (µV)	68.45 ± 6.24	70.25	4.69	54.32 ± 9.04	50.98	15.94
	Contra	P1 Latency (ms)	15.18 ± 0.09	15.20	0.10	15.54 ± 0.06	15.60	0.10
		N1 Latency (ms)	19.94 ± 0.54	20.10	0.90	21.00 ± 0.47	21.00	0.70
		P1-N1 Amplitude (µV)	67.09 ± 6.01	68.78	5.40	54.12 ± 9.35	51.20	11.95
AC	Ipsi	P1 Latency (ms)	18.98 ± 0.36	18.90	0.15	16.22 ± 0.34	16.20	0.20
		N1 Latency (ms)	25.95 ± 0.40	26.00	0.50	24.67 ± 0.39	24.60	0.40
		P1-N1 Amplitude (µV)	16.62 ± 3.81	16.43	2.45	45.55 ± 2.34	45.66	1.10
	Contra	P1 Latency (ms)	19.18 ± 0.63	19.20	0.82	16.26 ± 0.27	16.20	0.30
		N1 Latency (ms)	26.17 ± 0.33	26.25	0.47	25.10 ± 0.47	25.10	0.80
		P1-N1 Amplitude (µV)	11.66 ± 3.67	10.70	4.76	34.38 ± 1.54	35.20	2.40

### Bone Conduction mVEMP- Between Group Comparison

**Fig. 2:**
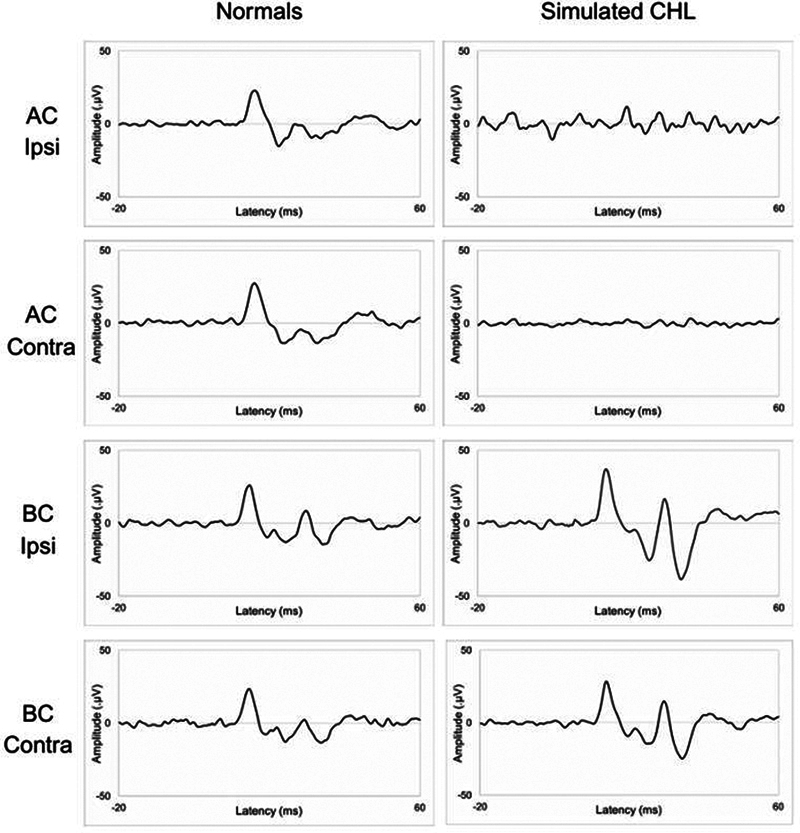
Grand-averaged mVEMP waveforms of masseter vestibular evoked myogenic potential (mVEMP) for the air conduction (AC) and bone conduction (BC), ipsilateral and contralateral responses for normal (NL) and simulated conductive loss (SC) groups.

A Mann-Whitney U test was conducted to compare the BC mVEMP measures between the NL and SC groups. BC responses were successfully elicited in all 50 participants across both groups ( Fig. 2 ). The SC group demonstrated significantly earlier latencies and higher amplitudes in both ipsilateral and contralateral conditions compared with the NL group. For ipsilateral recordings, the P1 latency was considerably earlier in the SC group (U = 173.00, p < 0.01, rrb = -0.45). Similarly, the SC group's N1 latency was significantly earlier (U = 81.00, p < 0.001, rrb = -0.74). The P1-N1 amplitudes were markedly greater in the SC group compared with the NL group (U = 560.00, p < 0.001, r_rb = 1.00).

A consistent pattern was observed for contralateral recordings. The SC group showed significantly earlier P1 latency (U = 0.00, p < 0.001, rrb = -1.00) and N1 latency (U = 43.00, p < 0.001, rrb= -0.86) relative to the NH group. Additionally, the P1-N1 amplitude in the SC group was significantly greater (U = 535.00, p < 0.001, rrb = 1.000).

### Air Conduction vs Bone Conduction mVEMP Responses in the NL Group

In the NL group, ipsilateral P1 latency was significantly shorter with BC stimulation (Z = -4.37, p < 0.001, r_rb = 1.000)x̀, while N1 latency was prolonged considerably in the AC modality (Z= -4.372, p <0.001, rrb= -1.000). However, no statistically significant difference was observed in the P1-N1 amplitude (Z= -0.34, p = 0.74, rrb= -0.08). For contralateral responses, similar findings were observed: P1 latency (Z = -4.37, p <0.001, rrb = -1.00) and N1 latency (Z= 4.37, p <0.001, rrb= 1.00) were significantly earlier with BC stimulation. Additionally, no significant differences were observed in the P1-N1 amplitude between the two modalities (Z = 1.14, p = 0.22, rrb = 0.27).

### Air Conduction vs Bone Conduction mVEMP Responses in SC Group

A significant difference was observed between the AC and BC for ipsilateral responses. In the SC group, P1 latency was significantly shorter with BC stimulation (Z = 2.20, p = 0.03, r_rb = 1.000), while N1 latency was also significantly shorter (Z = -2.20, p = 0.04, r_rb = -1.000). However, the P1-N1 amplitudes were significantly larger in the BC condition (Z= -2.20, p= 0.03, rrb= -1.00). For contralateral responses, similar findings were observed; the P1 (Z= -2.20, p= 0.04, rrb= -1.00) and N1 latencies (Z= 2.20, p= 0.031, rrb= 1.00) were significantly earlier with BC stimulation. Additionally, the P1-N1 amplitude was significantly greater for the BC (Z= 2.20, p= 0.03, rrb= 1.00). However, since the AC mVEMPs were obtained in 6 subjects, the statistical analysis should be interpreted cautiously, considering the possibility of type II errors and effect size inflation.

### Spectral Analysis of the Grand Average Waveforms

**Fig. 3:**
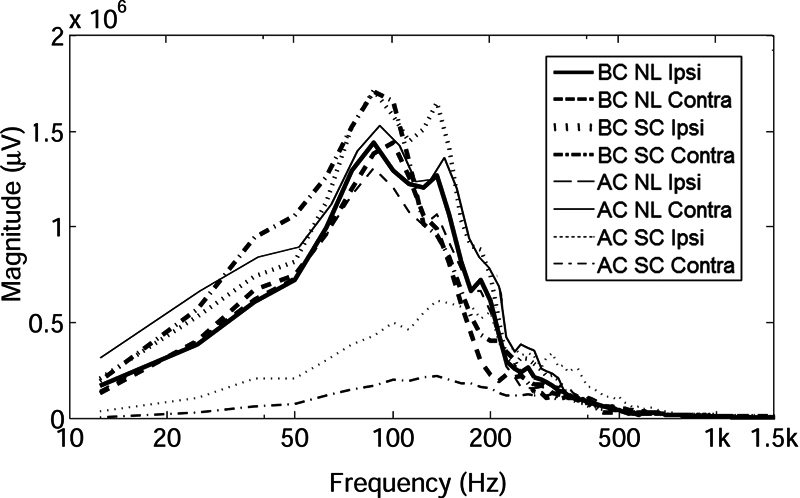
Spectral amplitudes for air conduction (AC) and bone conduction (BC) stimuli in normal (NL) and simulated conductive loss (SC) groups, recorded ipsilaterally (Ipsi) and contralaterally (Contra).

Fig. 3 illustrates the frequency-domain analysis of the grand average mVEMP responses obtained in the present study. Across all conditions, the spectral magnitude exhibited a prominent peak near 100 Hz. The 100 Hz spectral peak was most pronounced, particularly in the BC responses of SC groups. In contrast, air-conducted mVEMP responses in the SC group showed significantly reduced spectral energy, indicating attenuated responses due to conductive hearing loss simulation. The spectral profiles generally followed a band-pass characteristic with rising amplitude from ∼20 Hz, peaking near 80–100 Hz, and tapering off beyond 200 Hz. Notably, the BC responses exhibited greater magnitudes and broader spectral peaks than AC responses across both normal and SC groups, suggesting more robust activation via bone conduction.

## Discussion

The present findings provide compelling evidence that BC mVEMPs are significantly more prevalent and robust in the SC group than AC mVEMPs. This underscores the critical clinical utility of BC stimulation in situations where middle ear pathologies compromise conventional AC VEMP assessment.

### Stimulation Modes and mVEMP Responses

All 25 NL participants showed robust responses, confirming reliable vestibular activation via intact middle ear pathways. Only 6 of 25 SC participants produced detectable responses, demonstrating severe middle ear transmission impairment that prevented adequate otolithic stimulation. 22 The SC group exhibited a mean air conduction threshold of 33 ± 4.89 dBHL, with values ranging from 29 to 38 dBHL in the right ear and 31.60 ± 5.03 dBHL, with values ranging from 27 to 37 dbHL, indicating substantial variability in the degree of conductive attenuation. The variability in attenuation translates to correspondingly large differences in the effective stimulus level reaching the vestibule, thereby explaining the heterogeneous response pattern within the SC group for AC mVEMP. The six responsive SC participants exhibited significantly prolonged P1-N1 latencies and reduced amplitudes compared with the NL group, consistent with reported VEMP alterations in middle ear pathology. 23 24 These delays reflect compromised AC transmission efficiency rather than vestibular dysfunction, as evidenced by preserved BC VEMP responses in conductive hearing loss. 25 The NL group maintained consistent P1-N1 waveforms with normal latencies and amplitudes bilaterally.

Unlike the AC mVEMP, BC mVEMP responses were consistently recorded in all participants, including those in the simulated SC group. This indicates that BC stimuli directly bypass the middle ear and stimulate the vestibular system, making BC mVEMP a valuable alternative in cases where AC responses are absent or diminished. The observed BC mVEMP findings in this study, specifically the earlier P1 and N1 latencies and the larger P1-N1 amplitudes in the SC group compared to the NH group, are consistent with previous research and can be attributed to the occlusion effect. 15

The occlusion effect refers to the enhancement of BC sound perception when the external auditory canal is occluded, leading to increased sound pressure level, particularly at low frequencies such as 500 Hz. These phenomena result in more robust vestibular system stimulation, eliciting stronger VEMP responses. Studies have demonstrated that occluding the external auditory meatus can significantly increase the amplitudes of BC VEMPs. For instance, Betito et al. found that BC cVEMP amplitudes were substantially higher in occluded conditions compared with open ear canal conditions, highlighting the impact of the occlusion effect on vestibular responses. 15

Furthermore, earlier latencies observed in the SC group may be due to the enhanced transmission of BC stimuli resulting from the occlusion effect, which facilitates quicker activation of vestibular afferents. This is supported by Damien et al., who reported higher BC cVEMP amplitudes in children with middle ear effusion, underscoring the utility of BC stimulation when the AC pathway is compromised. 26 Additionally, Iwasaki et al. reported that BC-evoked responses were preserved in patients with middle ear disorders, further supporting the clinical utility of BC mVEMP in vestibular assessment.

Further, across both NL and SC groups, the ipsilateral and contralateral mVEMP responses exhibited remarkably similar trends in latency and amplitude, particularly under BC stimulation. This bilateral similarity is expected, as BC has an interaural attenuation of approximately 0 dB, allowing equal stimulation of vestibular structures on both sides. Consequently, BC likely activates both the ipsilateral and contralateral vestibular pathways simultaneously, resulting in highly comparable mVEMP latencies and amplitudes across ears. 27

### Spectral Analysis of mVEMP Responses

The observed spectral peak at approximately 100 Hz across all VEMP recordings corroborates prior findings. Singh et al. demonstrated, via power spectral analysis of ocular VEMPs, that most response energy resides below 500 Hz, with a spectral maximum centred around 100 Hz. 16 This spectral signature was consistent regardless of filter settings, supporting the physiological relevance of this frequency band for vestibular reflex activity. Lütkenhöner, using convolution models of cervical VEMP cVEMP signals, also reported that the power spectral density of EMG-derived responses peaks in the same ∼100 Hz range. 17 These results reinforce the interpretation that mVEMPs, like oVEMPs and cVEMPs, share similar frequency characteristics, likely reflecting the synchronized firing of motor unit potentials in response to vestibular input. The spectral analysis affirms the utility of ∼100 Hz as a biomarker frequency for VEMP analysis, and further studies are required to explore its possible diagnostic advantages in clinical vestibular assessments.

## Conclusions

The findings suggest that mVEMP responses, particularly those elicited via bone conduction, may offer more consistent and reliable results in conductive pathology than air conduction stimulation. AC mVEMP responses were absent or, if present, significantly reduced in amplitude and delayed in latency in the simulated conductive group, whereas BC responses remained robust. Additionally, the spectral analysis revealed a consistent peak around 100 Hz across conditions, similar to patterns observed in oVEMP and cVEMP studies. While this spectral feature in mVEMPs has not been widely reported, it may guide future parameter optimization.

## References

[1] Colebatch J G Halmagyi G M Vestibular evoked potentials in human neck muscles before and after unilateral vestibular deafferentation Neurology 1992 42 08 1635 1636 1641165 10.1212/wnl.42.8.1635

[2] Nagarajan A Sinha S K Masseter Vestibular evoked myogenic potentials: A new tool to assess the vestibulomasseteric reflex pathway J Otol 2024 19 01 46 54 38313757 10.1016/j.joto.2023.12.005 PMC10837556

[3] Rosengren S M Govender S Colebatch J G Ocular and cervical vestibular evoked myogenic potentials produced by air- and bone-conducted stimuli: comparative properties and effects of age Clin Neurophysiol 2011 122 11 2282 2289 21550301 10.1016/j.clinph.2011.04.001

[4] Rajesh A Neupane A K Masseteric vestibular evoked myogenic potentials in vestibular neuritis: A case series Iran J Otorhinolaryngol 2024 36 05 619 625 39323501 10.22038/ijorl.2024.77538.3598 PMC11421766

[5] de Natale E R Ginatempo F Paulus K S Abnormalities of vestibular-evoked myogenic potentials in idiopathic Parkinson's disease are associated with clinical evidence of brainstem involvement Neurol Sci 2015 36 06 995 1001 25567081 10.1007/s10072-014-2054-4

[6] Liu X Zhang S Huang X Zhang Y Fan D Vestibular evoked myogenic potentials and their clinical utility in patients with amyotrophic lateral sclerosis Clin Neurophysiol 2019 130 05 647 654 30870800 10.1016/j.clinph.2019.01.023

[7] Basta D Todt I Ernst A Normative data for P1/N1-latencies of vestibular evoked myogenic potentials induced by air- or bone-conducted tone bursts Clin Neurophysiol 2005 116 09 2216 2219 16043396 10.1016/j.clinph.2005.06.010

[8] Neupane A K Bhagat H Bheda K Comparison of chirp versus tone burst- and click-evoked masseteric vestibular evoked myogenic potentials in normal-hearing adults Am J Audiol 2023 32 02 303 313 36917064 10.1044/2022_AJA-22-00155

[9] De Natale E R Ginatempo F Mercante B Vestibulo masseteric reflex and acoustic masseteric Reflex. Normative data and effects of age and gender Clin Neurophysiol 2019 130 09 1511 1519 31295720 10.1016/j.clinph.2019.05.021

[10] Ramesh K Thirunavukkarasu K Decoding age-linked masseter vestibular evoked myogenic potential changes in healthy, aging individuals Am J Audiol 2024 33 03 838 849 38843439 10.1044/2024_AJA-23-00264

[11] Vispute R Neupane A K Exploring the Relationship between Masseter and Cervical Vestibular-Evoked Myogenic Potentials in Young Adults with Hearing Thresholds Less Than or Equal to 15 dB HL J Am Acad Audiol 2023 34(9-10):192 198 37657489 10.1055/a-2165-0935

[12] Kumar C B Pushpanathan S Abdullah Y Patel N R Thontadarya S Comparison of air conduction and bone conduction masseter vestibular evoked myogenic potential between neurotypical young adults and individuals with conductive hearing loss Cureus 2024 16 09 e70267 39463641 10.7759/cureus.70267 PMC11512666

[13] Bath A P Harris N McEwan J Yardley M P Effect of conductive hearing loss on the vestibulo-collic reflex Clin Otolaryngol Allied Sci 1999 24 03 181 183 10384842 10.1046/j.1365-2273.1999.00234.x

[14] Han P Zhang R Chen Z Evaluation of ocular and cervical vestibular evoked myogenic potentials in a conductive hearing loss model J Otol 2016 11 04 192 197 29937829 10.1016/j.joto.2016.12.002 PMC6002615

[15] Betito R Green K Stephens D Effect of external ear canal occlusion on bone-conducted cervical VEMP responses Ear Hear 2021 42 03 629 63533141776

[16] Singh N Thirunavukkarasu K Kumar P Barman A Effects of variation in response filter on ocular vestibular-evoked myogenic potentials: A preliminary investigation J Indian Speech Hear Assoc. 2019 33 02 79

[17] Lütkenhöner B Deconvolution of the vestibular evoked myogenic potential using the power spectrum of the electromyogram Theor Biol Med Model 2015 12 21 26438301 10.1186/s12976-015-0018-x PMC4594959

[18] Frank T ANSI update: maximum permissible ambient noise levels for audiometric test rooms Am J Audiol 2000 9 01 3 8 10943020 10.1044/1059-0889(2000/003)

[19] Carhart R Jerger J F Preferred Method For Clinical Determination Of Pure Tone Thresholds J Speech Hear Disord 1959 24 04 330 345

[20] Thirusangu V P Sinha S K Effect of electrode montage on 500-Hz tone burst evoked masseter vestibular evoked myogenic potential Am J Audiol 2022 31 02 403 410 35537126 10.1044/2022_AJA-22-00016

[21] Love J Selker R Marsman M JASP: Graphical statistical software for common statistical designsJ Stat Softw2019 8802

[22] Curthoys I S Grant J W Burgess A M Pastras C J Brown D J Manzari L Otolithic receptor mechanisms for vestibular-evoked myogenic potentials: A review Front Neurol 2018 9 366 29887827 10.3389/fneur.2018.00366 PMC5980960

[23] Wang M C Lee G S Vestibular evoked myogenic potentials in middle ear effusion Acta Otolaryngol 2007 127 07 700 704 17573565 10.1080/00016480601002070

[24] Wang M C Liu C Y Yu E C Wu H J Lee G S Vestibular evoked myogenic potentials in chronic otitis media before and after surgery Acta Otolaryngol 2009 129 11 1206 1211 19863312 10.3109/00016480802620654

[25] Yang T L Young Y H Vestibular-evoked myogenic potentials in patients with otosclerosis using air- and bone-conducted tone-burst stimulation Otol Neurotol 2007 28 01 1 6 17106429 10.1097/01.mao.0000244367.62567.0d

[26] Damien M Wiener-Vacher S R Reynard P Thai-Van H Bone conduction cervical vestibular evoked myogenic potentials as an alternative in children with middle ear effusion J Clin Med 2023 12 19 6348 37834992 10.3390/jcm12196348 PMC10573357

[27] Håkansson B Jansson K F Tengstrand T VEMP using a new low-frequency bone conduction transducer Med Devices (Auckl) 2018 11 301 312 30233258 10.2147/MDER.S171369 PMC6134943

